# When fate hands you lemons: A moderated moderation model of bullying victimization and psychological distress among Chinese adolescents during floods and the COVID-19 pandemic

**DOI:** 10.3389/fpsyg.2023.1010408

**Published:** 2023-03-03

**Authors:** Yuchi Zhang, Xiaoyu Jia

**Affiliations:** ^1^Department of Educational Technology, School of Smart Education, Jiangsu Normal University, Xuzhou, China; ^2^College of Teacher Education, Southwest University, Chongqing, China

**Keywords:** bullying victimization, neuroticism, negotiable fate, psychological distress, COVID-19, disaster

## Abstract

**Background:**

Bullying is a major problem worldwide and has numerous detrimental effects on the mental health of victims. The link between bullying and psychological distress in adolescents is well known. However, few studies have analyzed the impact of combined interpersonal, peer, and cultural factors on psychological distress using a social-ecological system framework in Eastern countries. Negotiable fate is a cultural belief common in Eastern societies that suggests that people can negotiate with fate for more control by exercising personal agency within the limits of what fate has determined. This study examined the moderating effects of neuroticism and negotiable fate on the relationship between bullying victimization and psychological distress among Chinese adolescents. Moreover, human society commonly suffers from multiple disasters that lead to severe mental health problems. There are few empirical studies on the effects of bullying among adolescents in multiple disaster contexts. This study included participants who experienced floods and COVID-19 simultaneously in 2021.

**Materials and methods:**

We conducted a cross-sectional cluster sampling study from August 6 to 9, 2021, approximately 2 weeks after the start of the Zhengzhou City flooding and 7 days after the new wave of the COVID-19 outbreak in Zhengzhou City. The study included 1,207 participants (52.4% men, *n* = 633; *M*_age_ = 14.36, *SD* = 0.94) from a middle school in Zhengzhou City, China.

**Results:**

The results revealed that bullying was positively linked to psychological distress (*β* = 0.5.34, *p* < 0.001, [0.73, 9.95]). Neuroticism and negotiable fate significantly moderated the relationship between the effects of bullying and psychological distress (*β* = −3.58, *p* < 0.05, 95% CI [−6.12, −1.04]). Specifically, high neuroticism increased the risk of psychological distress in adolescents bullied before a disaster. High or low neuroticism and high negotiable fate buffered the link between bullying and psychological distress.

**Conclusion:**

This study showed that neuroticism and negotiable fate moderated the relationship between bullying victimization and psychological distress in Chinese students with COVID-19 and flood disasters. High negotiable fate and high or low neuroticism could help adolescents bullied in school to be immune from psychological distress in catastrophe. The results highlight the importance of considering the interplay between bullying, neuroticism, and a sense of controllable destiny when examining adolescents’ psychological distress.

## 1. Introduction

“Effort is controlled by the person, success is determined by fate (Mou shi zai ren, cheng shi zai tian: “谋事在人, 成事在天”)” [Chinese proverb].

Bullying, a subtype of aggression, is characterized by repeated, deliberate, and imbalanced power and is a significant concern for schools and societies worldwide (Hong and Espelage, 2012; Hymel and Swearer, 2015). The prevalence of bullying among adolescents in the US and China is 11 and 17.3%, respectively (Hong and Espelage, 2012; Xue et al., 2022). Bullying is associated with mental health problems (Sigurdson et al., 2015; Baiden et al., 2019; Hysing et al., 2021). Previous studies have found that bullied adolescents in the US were twice as likely to have severe psychological distress compared to their non-victim peers (Zhang et al., 2016). Numerous studies have explored the relationship between bullying and psychological distress (Gong et al., 2020; He et al., 2021; Chen et al., 2022). Many antecedent variables have been identified as reducing or increasing the risk of psychological distress, such as personality type and social cognition (Gong et al., 2020; He et al., 2021; Chen et al., 2022).

There are still several gaps that need to be filled. First, most existing studies focus on the direct effects of personality on psychological distress (Gong et al., 2020; He et al., 2021; Chen et al., 2022). Very few have explored the potential underlying processes of personality traits on the links between bullying and psychological distress. Diathesis-stress models have shown that adolescents can experience negative health outcomes through interactions between individual characteristics (diatheses; neuroticism in the present study) and environmental effects (stressors; bullying in the present study) (Monroe and Simons, 1991; Ladd and Troop-Gordon, 2003). As peer relations play a vital role in adolescence, bullying is a negative life event (Hong and Espelage, 2012). Adolescents bullied at school may have higher baseline emotional or psychological problems and may be more susceptible to psychological distress (Glassner and Cho, 2018; Hysing et al., 2021; Norrington, 2021). Neuroticism (emotional instability) refers to “*the tendency to experience negative emotional instability*” (Liu et al., 2018) and is closely related to adolescents’ psychological distress (Liu et al., 2018; He et al., 2021; Chen et al., 2022). Thus, the first research question was: Does neuroticism moderate the direct effects of bullying in school on psychological distress among adolescents? Second, most studies on bullying victimization have been conducted in Western social contexts (Christina et al., 2021; Imuta et al., 2022; Polanin et al., 2022). To deepen our understanding of bullying victimization, it is necessary to examine the outcomes of bullying victimization in schools from a non-Western context. Chinese societies are deeply influenced by collectivistic and Confucian philosophy, which stresses interpersonal harmony and interdependence (Lim, 2009; Bond, 2010; Tom et al., 2010; Tong et al., 2022). Obtaining peer group approval and inclusion is especially vital for Chinese adolescents’ social lives (Lu et al., 2018; Tong et al., 2022). Thus, bullying victimization may present an urgent threat and become a negative life event for Chinese adolescent victims. Therefore, the second research question is: What are the effects of bullying victimization in schools in Chinese sociocultural contexts?

Third, not all bullied adolescents with high neuroticism experience the same level of psychological distress, and the more complex underlying processes are unclear. Specifically, the potential role of this mechanism in cultural factors has not been thoroughly examined in non-Western cultures. According to social-ecological system theory, children’s developmental outcomes result from the complex interactions of different ecological system-level factors (Bronfenbrenner, 1979). Microsystems and peer factors, such as bullying victimization, may interact with individual factors (i.e., neuroticism), and macrosystem factors (cultural values or beliefs) may further moderate the moderating effects of neuroticism on the associations between bullying victimization and adolescents’ mental health outcomes (Bronfenbrenner, 1979; Hong and Espelage, 2012). Negotiable fate refers to “the belief that individuals can negotiate with fate for control, and they do this by exercising personal agency within the limits that fate has determined.” (Au et al., 2012). This is a common cultural belief, particularly in Eastern cultures (Au et al., 2011, 2012). The concept of this belief can be epitomized by the popular Chinese saying, “Man proposes, fate disposes” (Mou shi zai ren, cheng shi zai tian). A similar saying in Western culture states: “If fate hands you lemons, make lemonade.” Negotiable fate plays a vital role in coping strategies and well-being. To the best of our knowledge, no empirical studies have examined the intricate moderating effects of individual (neuroticism), microsystem (bullying victimization), and macrosystem (negotiable fate) factors on the mental health outcomes of adolescents. This lack of research limits the advancement of comprehensive and culturally-sensitive theories and school-based mental health interventions. The third research question was: Does negotiable fate moderate the moderating effects of neuroticism on the association between bullying and psychological distress?

To fill these gaps, this cross-sectional study aimed to merge the diathesis-stress models with social-ecological system theory and examine the moderating role of neuroticism and the moderated moderating role of negotiable fate (three-way interaction effect) in the relationship between bullying and Chinese adolescents’ psychological distress.

### 1.1. Bullying victimizations and psychological distress

Adolescence is a critical period of transition to adulthood that is characterized by fluctuating levels of maturity and pivotal for growth into a well-adjusted and healthy adult (Steinberg, 2005; Sawyer et al., 2018; Halliday et al., 2021). Adolescence is also a period of vulnerability to psychological distress, and the effects of traumatic experiences during these periods may have long-term negative consequences on individuals’ mental health and well-being in adulthood (Halliday et al., 2021).

Bulling victimization is a high prevalence school mental health concern worldwide (Arseneault, 2018; Sampasa-Kanyinga et al., 2018; Halliday et al., 2021; Jadambaa et al., 2021; Norrington, 2021; Salmivalli et al., 2021; Islam et al., 2022; Serafini et al., 2023). It has a series of negative effects on adolescents’ physical and mental health outcomes (Arseneault, 2018; Norrington, 2021; Islam et al., 2022). In the short term, being bullied in school could lead to poorer sleep quality and academic outcomes, higher stress, depression, anxiety levels, and higher risk of suicide attempts (Jadambaa et al., 2020; Halliday et al., 2021). A meta-analysis research found strong causal associations between adolescents’ bullying victimization and mental health problems (e.g., anxiety, depression, poor general health, and suicidal ideation) (Moore et al., 2017). There is growing longitudinal evidence suggesting that being bullied during adolescence is significantly associated with mental health problems in adults (Roberts et al., 2013; Matthews et al., 2022). For example, a longitudinal Norwegian study showed that being bullied in the middle-school positively predicted internalizing problems 12 years later (Sigurdson et al., 2015). A meta-analysis of 66 studies proved that bullying victimization in adolescence could increase the risk of developing internalizing problems (depression and anxiety symptoms) and suicidal ideation across the lifespan (Zych et al., 2015). However, most studies were conducted in Western cultural contexts, which provide a more comprehensive understanding of bullying victimization in different cultures. Thus, the present study attempts to examine the relationship between bullying victimization and psychological distress in Chinese adolescents.

### 1.2. The moderating role of neuroticism

Given the deleterious effects of school bullying victimization on psychological distress among adolescents, understanding the potential underlying processes by which bullying victimization may have a stronger or weaker impact on psychological distress is pivotal to developing effective tailored intervention programs. Nevertheless, studies focusing on the moderating role of the association between bullying victimization and psychological distress in non-Western cultures are limited. The diathesis-stress model posits moderating effects of personality on stressful circumstances (Monroe and Simons, 1991; Ladd and Troop-Gordon, 2003). Adolescents with high neuroticism commonly have higher emotional instability, tend to negatively process emotion-related information, and prefer to adopt fewer positive coping strategies during negative life events (Su et al., 2018; Wang et al., 2019). Similarly, the social-ecological system theory suggests that adolescents’ outcomes are the result of the effects of interaction between individual factors (neuroticism) and the contexts in which they live (e.g., microsystem factors, such as peers and family; macrosystem factors, such as cultural beliefs) (Bronfenbrenner, 1979). A previous study found that low neuroticism (high emotional stability) significantly moderated the positive association between exposure to violence and internalizing problems among Chinese adolescents (Ho et al., 2013). Based on the diathesis-stress model and social-ecological system theory, we propose that neuroticism can moderate the association between bullying victimization and psychological distress among Chinese adolescents. Specifically, adolescents with high neuroticism may have poorer personal resources to regulate negative emotions and cope with negative life events in the environment; they may tend to perform more maladaptive emotional regulation and adopt passive coping strategies when bullied in school (Nelis et al., 2011). Consequently, their depression, anxiety, and stress levels may surge when they become victims of bullying incidents. A previous study found that higher exposure to violence and high levels of neuroticism were positively associated with symptoms of depression and anxiety (Ho et al., 2013).

### 1.3. Moderated moderating effect of negotiable fate

Most studies on bullying victimization and mental health have considered the interaction between individuals and contextual factors (family, peer, and school factors). However, to the best of our knowledge, none of the empirical studies has fully considered the potential moderating role of cultural factors and further explored more complex interactions (three-way interaction effect). Adolescents’ psychological distress is a consequence of complex interactions between individual factors and the contexts in which they live, drawing on the social-ecological system theory (Bronfenbrenner, 1979). When individuals enter adolescence, the macro system becomes momentous (Tynan et al., 2015). The social-ecological system theory and researchers studying bullying emphasize the critical role of the macrosystem (i.e., negotiable fate in the present study) (Ladd and Troop-Gordon, 2003; Hong et al., 2016). Nonetheless, very few empirical studies have tested the role of cultural beliefs in bullying victimization, let alone the complex interaction effects between different system levels on the associations between bullying victimization and adolescents’ psychological distress.

The cultural belief of fatalism is the belief that an individual’s life outcomes are fully pre-determined by fate and that an individual’s actions cannot change the future (Zhang et al., 2021). Contrastingly, negotiable fate believes that “fate imposes boundaries within which personal actions can shape outcomes” (Au et al., 2017). Although these beliefs exist in both Eastern and Western general groups, they are quite common, have a significant role in Chinese society, and are closely related to individuals’ active coping strategies and positive self-views (Au et al., 2012). Based on the social-ecological system theory, the present study assumed that the moderating effect of neuroticism (individual-level factors) on the relationship between bullying victimization (microsystem-level factor) and psychological distress might be affected by belief in negotiable fate (macrosystem-level factor). Conversely, adolescents who endorsed negotiable fate tended to acknowledge the constraints (e.g., negative life events) they experienced as fate but without being pessimistic; at the same time, they believed that even though these constraints could not be overcome, they still actively found ways to pursue their personal goals under the constraints of fate (Au et al., 2012). Thus, even if adolescents are bullied in school, they may perceive bullying as a fate constraint. The negative emotional fluctuation caused by high neuroticism may also be perceived as “fates’ constraints.” Thus, when adolescents with high neuroticism and a strong belief in negotiable fate are bullied, they adopt more active coping strategies to regulate their emotional states and social life (Au et al., 2012, 2017). As a result, bullying victimization may not induce higher levels of psychological distress. If adolescents strongly believe in negotiable fate but have low neuroticism (high emotional stability). In that case, they may have richer resources to support their ability to regulate their emotions and actively resolve their problems in school (Nelis et al., 2011). Thus, adolescents with high negotiable fate and high or low neuroticism may be immune to the bullying victimization effect.

Conversely, adolescents with a weak sense of negotiable fate but high neuroticism may deny that their actions still matter despite uncertainty factors that they could not control (such as the COVID-19 pandemic or being bullied in school). Concurrently, they also tend to adopt more maladaptive emotion regulation skills and have difficulty maintaining well-functioning mental states (Auerbach et al., 2010; Hampel and Petermann, 2020; Yang et al., 2020). Adolescents who are bullied at school may consider their experience as foreordained and assume that they cannot change it. This can cause them to have a higher risk of adopting passive or maladaptive coping strategies, which may result in increased depression, anxiety, and stress (Hampel and Petermann, 2020).

Exploring the moderated moderating effects (three-way interaction effect) of negotiable fate and neuroticism on the association between bullying and psychological distress could contribute to our understanding of bullying and psychological distress from a social-ecological system perspective.

### 1.4. The Chinese social culture context

Prior studies on peer factors and adolescents’ psychological distress have focused on Western cultures (Steinberg, 2005; Sawyer et al., 2018; Islam et al., 2022). Few studies have shown positive links between bullying victimization and psychological distress in non-Western contexts (Sampasa-Kanyinga et al., 2018).

There are three main implications for exploring bullying victimization and adolescents’ psychological distress in Chinese social culture contexts. First, negotiable fate is deeply rooted in the wisdom of traditional Chinese culture and there is significant theoretical value in examining its role in modern China (Au et al., 2011, 2012). Researchers imply that the belief in negotiable fate is more prevalent in China than in the United States because ancient China was traditionally disaster-prone and frequently experienced social turbulence, which made individuals uncertain about their ability to control their lives (Au et al., 2011, 2012, 2017). Endorsing negotiable fate helps individuals be more active in striving to achieve their goals under uncertainty. However, Chinese people who simply believed in fatalism or fully emphasized self-determination were more likely to suffer from long-term psychological pain and dysfunction. China has developed rapidly in recent decades in terms of economy and globalization and is heavily influenced by Western culture. It is urgent to explore whether negotiable fate can still protect individuals from negative life events (being bullied, disasters). Second, Chinese culture places high value on interpersonal interdependence and the maintenance of harmony with others. Therefore, being bullied by peers is a more culturally threatening event for Chinese adolescents (Tong et al., 2022). It is ideal to explore the specificity of the role of being bullied in a collectivist culture. Third, the coronavirus disease 2019 pandemic (COVID-19) has caused serious and profound psychological distress in children and adolescents in China and other places worldwide (Deolmi and Pisani, 2020; Chawla et al., 2021). Adolescence is a period of life that is characterized by high vulnerability to psychological distress. COVID-19 caused psychological distress among adolescents in Western and Eastern societies (Mazza et al., 2020; Qin et al., 2021). Moreover, like other countries, China often suffers from multiple disasters simultaneously. Preliminary studies on disasters may provide unparalleled information for a fruitful understanding of psychological distress. At the end of July 2021, Zhengzhou City in China suffered dangerous flooding, which killed 302 people and affected nearly 14.53 million people. The new wave of COVID-19 hit Zhengzhou before the flooding ended, causing a “double disaster.” The local government requested citizens to stay home or quarantine themselves (China Daily, 2021; Henan Provincial Government, 2021). Thus, China may provide unique contexts for investigating the association between bullying victimization, neuroticism, negotiable fate, and adolescents’ psychological distress during disasters.

### 1.5. The current study

**Figure 1:**
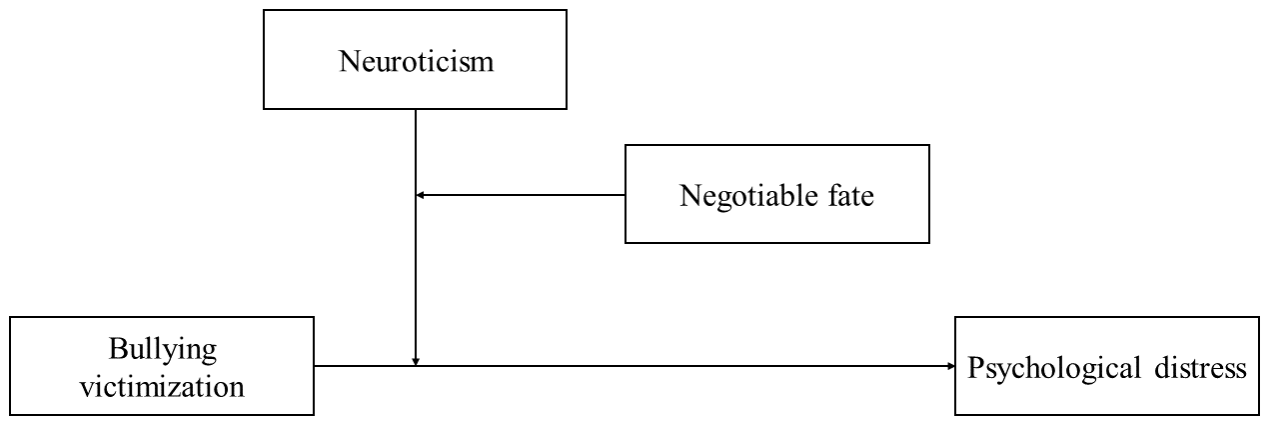
Hypothesized conceptual model.

This study aimed to explore the influence of bullying victimization on Chinese adolescents’ psychological distress among using a moderated moderation model with a cross-sectional design. The participants were adolescents in Zhengzhou City, China, who experienced floods and COVID-19 simultaneously at the beginning of August 2021. As shown in the hypothesized model (Figure 1), this study explored whether the positive links between bullying victimization in school and adolescents’ psychological distress during disasters were moderated by neuroticism and whether negotiable fate moderated the moderating effects of neuroticism in three-way interaction effect. This study proposed the following hypothesis:

*Hypothesis 1*: Adolescents’ bullying victimization was positively correlated with psychological distress.

*Hypothesis 2*: Neuroticism moderated the positive relationship between bullying victimization and psychological distress among Chinese adolescents. Specifically, the positive relationship between bullying victimization and psychological distress was stronger for adolescents with high neuroticism.

*Hypothesis 3*: Negotiable fate moderated the moderating effects of neuroticism on the relationship between bullying victimization and psychological distress among Chinese adolescents. Specifically, the positive relationship between bullying victimization and psychological distress was not significant for adolescents with high negotiable fate and high or low neuroticism.

## 2. Materials and methods

### 2.1. Participants

This study was approved by the research ethics committee at the corresponding author’s institution following the Declaration of Helsinki. Data collection was conducted online from August 6 to 9, 2021, approximately 2 weeks after the start of the Zhengzhou flooding and 7 days after the new wave of COVID-19 outbreak in Zhengzhou City. Students were on summer vacation and not allowed to return to school due to the disasters. Thus, teachers were instructed to use social networking software (WeChat) to explain the purposes of this investigation and the participants’ rights. Parents and students were asked to provide informed consent online. Subsequently, participants were instructed and guided to the survey on a widely used online survey website in China.^1^ The inclusion criteria: the participants who were high school students and lived in Zhengzhou city during the flood and COVID-19 pandemic were recruited. The exclusion criteria were (Hymel and Swearer, 2015) being diagnosed with mental disorders and (Hong and Espelage, 2012) participants’ family members was confirmed with COVID-19 or died in the 2021 flooding.

The sample was collected through a cluster sampling method, and data were collected from 1,207 participants (52.4% men, *n* = 633; *M*_age_ = 14.36, *SD* = 0.94) from a middle school in Zhengzhou. Participants were from 7th to 9th grades (7th grade: *n* = 460; 8th grade: *n* = 414; 9th grade: *n* = 333). All participants were included in the study, as none of them met the exclusion criteria. All participants and their parents provided informed consent, and they were informed that they could voluntarily withdraw from the study at any stage. Data from three participants were eliminated because they responded to all research items uniformly. The final sample comprised 1,204 students (7th grade: *n* = 459; 8th grade: *n* = 414; 9th grade: *n* = 331).

### 2.2. Measures

#### 2.2.1. Bullying victimization

Bullying victimization experiences in schools were measured using an adapted version of the bullied scale (Sigurdson et al., 2015). Followed by previous studies procedure, all participants should read a instructions about the concept definitions of bullying in school before the answer the scale (repeatedly being physical assault, taking a nickname the other person does not like, being verbally abused, making malicious jokes, or deliberately ostracizing, isolating, spreading rumors, or taking things like money, or being bullied by any other intentional harm that the bullied student has trouble resisting on his or her own) (Kyriakides et al., 2006). Participants rated the items on a five-point Likert scale (0 = never; 1 = 1–2 times; 2 = about once a week; 3 = 2–3 times a week; 4 = more often) to report their bullying experience (included three main traditional bullying type in China: physical, verbal, and relational bullying) in school during the last 6 months (last semester, before the flood disasters begin). As many studies have used categorical variables to measure bullying victimization, the procedures of previous studies were followed to make the results of this study comparable with those of previous studies (Guy et al., 2017; Husky et al., 2020; Lin et al., 2020; Eyuboglu et al., 2021; Hysing et al., 2021; Lin et al., 2022). Responses were recorded as 0 (rated 0, no response) or 1 (Hong and Espelage, 2012; Hymel and Swearer, 2015; Hysing et al., 2021; Xue et al., 2022).

#### 2.2.2. Neuroticism

The neuroticism subscale of the ten-item version of the Big Five Inventory (BFI-10) was adopted to measure participants’ neuroticism (Rammstedt, 2007), which included two items rated on a five-point Likert scale (1 = strongly disagree; 5 = strongly agree) (sample item: “I often worry about trifles”). Higher scores indicate greater neuroticism. This scale has been widely used in Western and Chinese samples (Rammstedt, 2007; Carciofo et al., 2016), and has shown good reliability and validity in Chinese samples (Carciofo et al., 2016).

#### 2.2.3. Negotiable fate

Negotiable fate was measured using an adapted version of the negotiable fate questionnaire with four items rated on a six-point Likert scale (1 = strongly disagree; 6 = strongly agree) (sample item: “If I put in the effort to do the best I can, fate will take care of the rest.”) (Au et al., 2011, 2012). Higher scores indicated a stronger belief in negotiable fate. This questionnaire has been translated and used in previous Chinese research and has good reliability and validity (Zhang et al., 2021). The Cronbach’s alpha for this study was 0.88.

#### 2.2.4. Psychological distress

The adaptive version of 21-item Depression, Anxiety, and Stress Scale (DASS-21) was used to measure psychological distress during COVID-19 and flood (Lovibond and Lovibond, 1996). The DASS-21 is suitable for non-clinical adolescent populations (Henry and Crawford, 2005). The participants were asked to rate the frequency and/or severity of symptoms since the Zhengzhou flood on a four-point Likert scale (0 = did not apply at all; 3 = applied very much or most of the time) (sample items: Depression: “wasn’t worth mush”; Anxiety: “trembling”; Stress: “intolerant”). Higher scores indicated higher psychological distress. The translated Chinese version showed good reliability and validity in a previous study (Zhang et al., 2021). The Cronbach’s alpha for this study was 0.94.

### 2.3. Covariate variables

This study used sex (male = 1, female = 2) and grade (grades 7–9) as covariate variables, as these could be related to psychological distress (Van Droogenbroeck et al., 2018).

### 2.4. Data analysis

Data analyses were performed using SPSS 22.0 (SPSS Inc., Chicago, IL, United States). First, we conducted a common method bias test to ensure that the data met the general requirements of the analysis. Descriptive statistics and correlation analyses of the variables were performed to obtain a general overview of the studied associations. Second, confirmatory factor analysis (CFA) was performed to construct the measurement model to examine the validity of variables using AMOS 23. We evaluated the goodness of fit of measurement and structural models using chi-square (df), goodness-of-fit index (GFI), incremental fit index (IFI), comparative fit index (CFI), and root mean square error of approximation (RMSEA) (Hu and Bentler, 1998). Third, to test our hypotheses, we employed PROCESS macro 3.0, nested in SPSS, which has been widely used to test mediation and moderated mediation models (PROCESS Models 1 and 3) (Preacher et al., 2007). Repeated sampling tests with 5,000-time bootstraps were used to test the significance of the effect. If the 95% confidence interval (CI) did not include 0, the effect was considered significant (Preacher et al., 2007). Parameter estimates for pathways that were statistically significant if *p* < 0.05 (two-tailed).

## 3. Results

### 3.1. Preliminary analyses

First, Principal component analysis on all variables extracted 5 eigenvalues greater than 1. The first factor explained 36.02% of the variance, which was less than the critical value of 40%, demonstrating that the common method bias was not a significant problem in the current study (Preacher et al., 2007).

Second, CFA model fit indices showed that the model fit the data well: *χ*^2^ = 1506.28, *df* = 436, *p* < 0.001, *χ*^2^/*df* = 3.46, GFI = 0.92, IFI = 0.95, RMSEA = 0.05, 90% confidence interval (CI) = (0.04, 0.05). All standardized factor loadings were significant (*p* < 0.001), and none of the factor loadings were smaller than 0.45 (Hu and Bentler, 1998).

Third, the means, standard deviations, and correlation matrices of the variables revealed a significant positive correlation between bullying victimization, neuroticism (*r* = 17, *p* < 0.001), and psychological distress (*r* = 21, *p* < 0.001; Table 1). Negotiable fate was significantly and negatively correlated with neuroticism (*r* = −0.26, *p* < 0.001) and psychological distress (*r* = −0.26, *p* < 0.001); however, the correlations with bullying victimization were non-significant (*r* = −0.03, *p* > 0.05).

**Table 1:** Descriptive statistics and correlations among variables.

	1	2	3	4	5	6
1. Bullying victimization	1					
2. Neuroticism	0.17^**^	1				
3. Negotiable fate	−0.03	−0.26^**^	1			
4. Psychological distress	0.21^**^	0.38^**^	−0.18^**^	1		
5. Sex (Male = 1)	0.02	0.11^**^	0.01	0.11^**^	1	
6. Grade	−0.03	−0.05	0.04	−0.05	−0.03	1
*M*	0.07	4.83	4.68	16.56	54.4	-
*SD*	-	1.65	0.95	18.23	-	-
Min-max	0–1	2–10	1–6	42–168		

Sex is coded as 1 = Male, 2 = Female. Grade is coded as 1 = Grade 10th, 2 = Grade 11th, 3 = Grade 12th. The mean for child sex reflects the percentage of male students; the mean for bullying victimization reflects the percentage of being bullied students. *p < 0.05; **p < 0.01.

### 3.2. Testing for the moderation model

**Figure 2:**
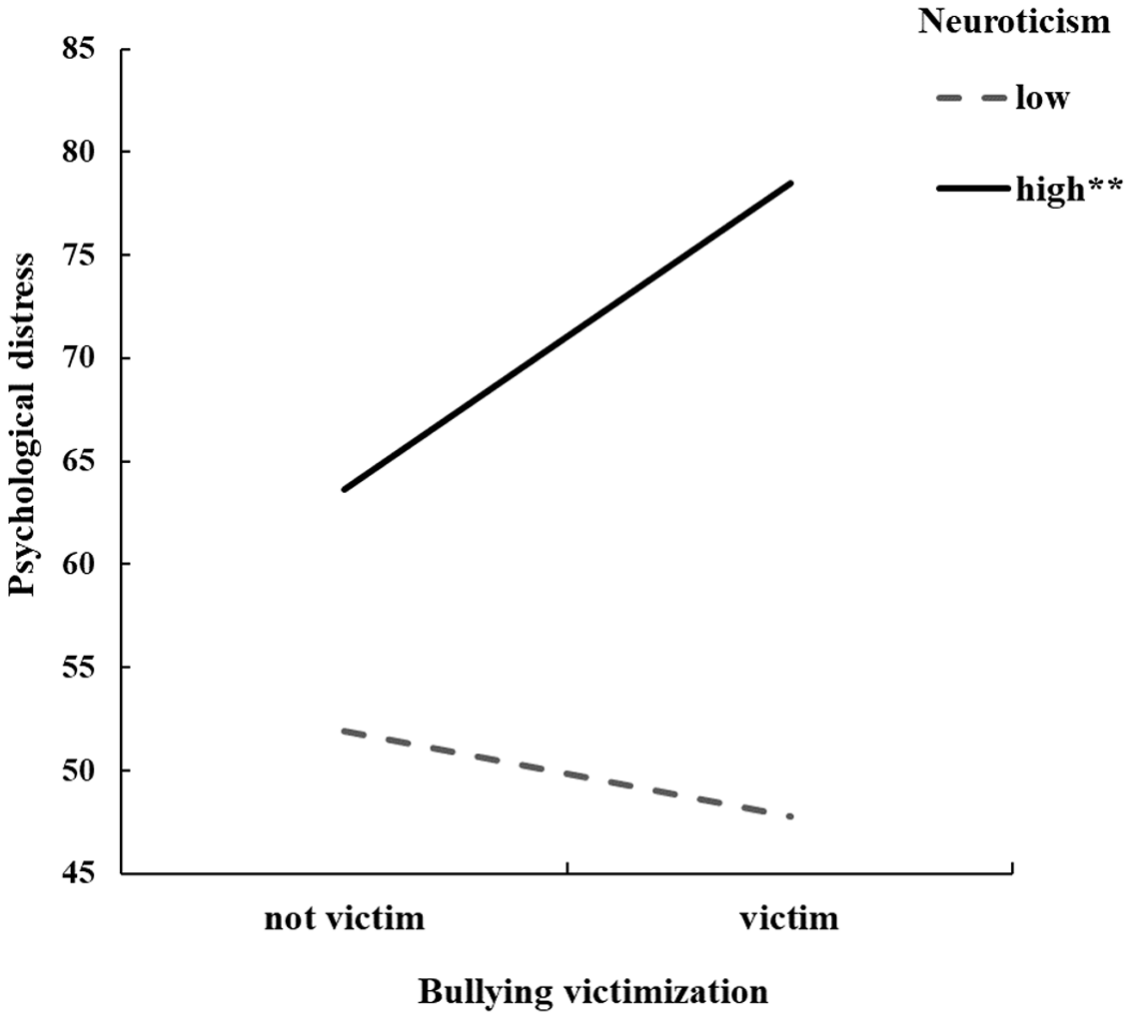
Interaction between bullying victimization and neuroticism in predicting psychological distress among adolescents. The y-axis of Figure 2 does not start at 0.

Model 1 in the SPSS macro PROCESS 3.0, compiled by Hayes, was used to compute the moderation model (Hayes, 2017). After controlling for gender and grade, bullying victimization during the double disaster was significantly positively related to adolescents’ psychological distress (*β* = 0.5.34, *p* < 0.001, [0.73, 9.95]). Neuroticism significantly moderated the positive link between bullying victimization and psychological distress (*β* = 5.75, *p* < 0.001, [3.18, 8.32]). Simple slope analysis indicated that the relationship between bullying victimization and psychological distress varied as a function of the degree of neuroticism. When neuroticism levels were high (one *SD* over the mean), the positive relationship between bullying victimization and psychological distress during disasters was significant (*b_simple_* = 14.83, *t* = 7.00, *p* < 0.001, 95% CI [10.67, 18.98]; Figure 2). When neuroticism was low (one *SD* below the mean), the relationship between bullying victimization and psychological distress was non-significant (*b_simple_* = −4.14, *t* = −1.04, *p* = 0.30, 95% CI [−11.95, 3.68]).

### 3.3. Testing for the moderated moderation model

**Figure 3:**
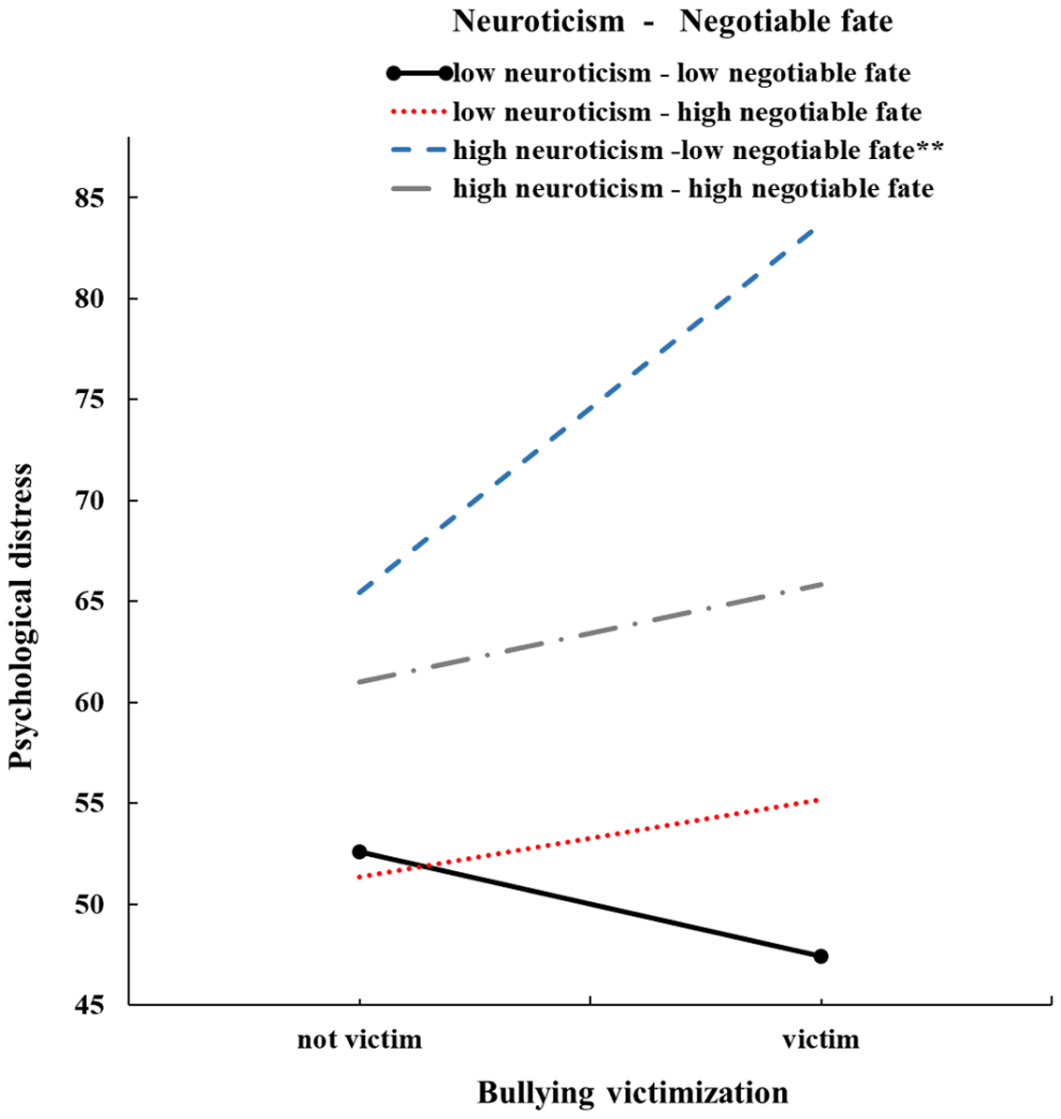
Three-way interaction effect of bullying victimization, neuroticism, and negotiable fate on adolescent depression. The y-axis of Figure 3 does not start at 0.

The SPSS macro PROCESS 3.0 (Model 3) was used to test the moderated moderation (three-way interaction) model (Table 2; Figure 3). The three-way interaction of bullying victimization × neuroticism × negotiable fate was significant (*β* = −3.61, *p* < 0.05, 95% CI [−6.15, −1.08]). The moderated moderating model explained 20% of the variance in adolescents’ psychological distress. Simple slope analyses indicated that the association between bullying victimization and psychological distress was not significant when negotiable fate was high, regardless of the level of neuroticism (1 SD above the mean; *b_simple_* = 4.86, *t* = 1.38, *p* = 0.17, 95% CI [−2.07, 11.80]). When negotiable fate was low and neuroticism was high (1 SD below the mean), the positive links between bullying victimization and psychological distress were significant (*b_simple_* = 18.28, *t* = 6.81, *p* < 0.001, 95% CI [13.02, 23.55]). When negotiable fate was high and neuroticism was low, the association between bullying victimization and psychological distress was not significant (1 SD below the mean: *b_simple_* = 3.79, *t* = 0.70, *p* = 0.49, 95% CI [−6.91, 14.50]). The results of the slope difference test are shown in Table 3. The association between bullying and psychological distress were significantly differ from others (Dawson and Richter, 2006).

**Table 2:** Model summary of the moderated moderating effect of neuroticism and negotiable fate in the associations between bullying victimization and psychological distress (*N* = 1,204).

Variables	*β*	*SE*	*t*	*p*	95% CI	
**Outcomes = psychological distress**						
Bullying victimization	5.44	2.34	2.32	0.00^**^	0.84	10.04
Neuroticism	3.55	0.30	11.68	0.00^**^	2.95	4.15
Negotiable fate	−1.62	0.53	−3.08	0.00^*^	−2.65	−0.59
Bullying victimization × Neuroticism	3.71	1.37	2.70	0.01^*^	1.02	6.40
Bullying victimization × Negotiable fate	−1.19	2.75	−2.79	0.66	−6.60	4.21
Neuroticism × Negotiable fate	−0.74	0.29	−2.52	0.01^*^	−1.31	−0.16
Bullying victimization × Neuroticism × Negotiable fate	−3.61	1.29	−2.79	0.00^*^	−6.15	−1.08
Sex (Male = 1)	2.62	0.95	2.77	0.00^*^	0.77	4.48
Grade	−0.39	0.59	−0.68	0.50	−1.55	0.76
*R* ^2^	0.21					
*F*	34.55^**^					

Sex coded as 1 = Male, 2 = Female. Grade is coded as 1 = Grade 10th, 2 = Grade 11th, 3 = Grade 12th. *p < 0.05; **p < 0.01. These values are based on unstandardized path coefficients. All parameter estimates and significance tests are based on 5,000 bootstrapped samples. Significant effects are determined by both 95% CI that does not include zero. Covariates included participants’ sex and grade.

**Table 3:** A significance test of differences between pairs of slopes for the bullying victimization on psychological distress.

Pair of slopes	*t*-value	value of *p*
(1) and (2)	−8.746	0.000^**^
(1) and (3)	−7.621	0.000^**^
(1) and (4)	−8.000	0.000^**^
(2) and (3)	−4.603	0.000^**^
(2) and (4)	−7.132	0.000^**^
(3) and (4)	−8.573	0.000^**^

(1) Stands for high neuroticism and high negotiable fate; (2) Stands for high neuroticism and low negotiable fate; (3) Stands for low neuroticism and high negotiable fate; and (4) Stands for low neuroticism and low negotiable fate. ^*^p < 0.05; ^**^p < 0.01.

## 4. Discussion

To our knowledge, this is the first empirical study to explore the effect and underlying processes of bullying victimization on adolescents’ psychological distress based on the ecological system theoretical framework. The results indicated that neuroticism and negotiable fate had significantly moderated moderating roles in the association between bullying victimization and psychological distress in Chinese adolescents who concurrently experienced floods and COVID-19.

### 4.1. Moderating effects of neuroticism

Bullying victimization was positively correlated with psychological distress during disasters, supporting *Hypotheses 1*. This was consistent with prior findings and extended by revealing the effect in an Eastern society (Zhang et al., 2016). Moreover, neuroticism moderated the positive link between bullying victimization and psychological distress, supporting *Hypotheses 2*. Our findings extended the social-ecological system theory and diathesis-stress model in disasters by showing that individual factors interacted with microsystem factors in adolescents’ psychological distress during multiple disasters (Monroe and Simons, 1991; Ladd and Troop-Gordon, 2003). According to the diathesis-stress model, neuroticism is a diathesis that increases the vulnerability of adolescents to stressful environments. Thus, being bullied and suffering from double disasters may induce psychological distress in these youth. Previous studies support this finding. Adolescents with high neuroticism who were bullied in school before the disaster had higher anxiety and tended to use more maladaptive coping strategies (Au et al., 2011). A related study found positive links between exposure to violence and internalizing problems such as anxiety and depression were stronger among Chinese adolescents with high neuroticism (Ho et al., 2013). Our findings are inconsistent with those of previous studies. Calvete et al. found that neuroticism failed to moderate the link between bullying victimization and internalizing symptoms among Spanish adolescents (Calvete et al., 2016). One possible reason for this inconsistency is that the dependent variable in our study differs from that in previous studies. Previous studies have defined the internalization problem as depression and social anxiety, rather than depression, general anxiety, and stress, as in this study.

### 4.2. The moderated moderating effect of negotiable fate

Importantly, this study demonstrated the protective role of cultural factors on adolescents’ psychological distress. Consistent with *Hypothesis 3*, this study found that Chinese bullying victims with highly negotiable fates buffered the risk-moderating effects of high neuroticism on the association between bullying victimization and psychological distress. These findings support and extend the social systems theory on bullying victimization research (Bronfenbrenner, 1979). Chinese adolescents who endorsed negotiable fate and experienced bullying tended to accept these negative life events (e.g., bullying victimization and the negative effects of emotional instability) as constraints on their fate. Furthermore, they believe they can actively find strategies to attain personal goals under these constraints (Au et al., 2011, 2012, 2017). As a result, they did not experience increased psychological distress when bullied. Adolescents with a weak sense of negotiable fate and high neuroticism may perceive negative life events and repeatedly fluctuating emotional states as uncontrollable “fate.” They may prefer to use more passive coping strategies and perform more biased and negative social information processes when they are bullied, which increases their psychological distress (Roelofs et al., 2008; Guy et al., 2017; Hampel and Petermann, 2020; Puechlong et al., 2020; Yang et al., 2020). Our findings highlight the importance of investigating the protective role of culturally specific factors in the association between bullying victimization and psychological distress. These findings illustrate the complex interactions between different levels of ecological system factors in adolescents’ mental health in the Chinese cultural context.

Additionally, this study was conducted while the participants were experiencing a double disaster and examined variables by measuring the participants’ reactions while still experiencing the disasters. Therefore, this study can explore potential antecedent variables and mechanisms that may be associated with psychological distress in adolescents during disasters. However, as we did not directly provide any data on psychological distress before or after disasters, the present study’s findings must be interpreted cautiously. They may only reflect associations in the general context and not in the disaster-specific model. This study provides preliminary evidence and directions for future research.

### 4.3. Strengths, limitations, and future directions

From the theoretical view, this study highlighted the protective role of cultural factors in bullying victim adolescents’ psychological distress. Moreover, this is the first empirical study to include individuals, microsystems, and macrosystems in non-Western cultures contexts. The findings could benefit the literature by providing evidence to build more cultural sensitivity and ecological system-based theory to explain adolescents’ psychological distress. From a practical perspective, these findings suggest that school counselors in China should prioritize bullying victims, especially those with high neuroticism and denial of negotiable fate, as intervention targets. School mental health prevention programs could train Chinese adolescents to avoid or reduce bullying risk. Moreover, they may benefit from an increased belief in negotiable fates. As neuroticism and cultural beliefs are both intervenable factors (Sauer-Zavala et al., 2017; Arieli and Sagiv, 2018), exploring their role in the association between bullying victimization and psychological distress will provide critical and useful information for school psychologists.

This study had several limitations. First, as it adopted a cross-sectional design, causal relationships between variables could not be determined. Future studies should adopt longitudinal designs. Second, as this study aimed to explore psychological distress among adolescents during multiple disasters, we recruited participants from one school, which may limit the generalizability of the results. Third, as this study adopted single self-reported measures to collect data, future research could benefit from using multiple sources of data, such as self-and peer-reports. Fourth, we did not distinguish between different types of bullying victimization in schools. Further research would benefit from exploring the unique effects of different types of bullying victimization, such as cyberbullying, during multiple disasters. Fifth, this study did not directly measure baseline levels of psychological distress in pre-disaster adolescents, which made it difficult to fully demonstrate that the model in our study reflects a specific pattern for a double disaster. Future studies should compare baseline levels of psychological distress before and after a disaster to answer this question (Kock et al., 2021).

## 5. Conclusion

This study confirmed and extended previous research by examining the association between bullying victimization in school and Chinese adolescents’ psychological distress, and determining the moderated moderating effect (three-way interaction) of neuroticism and negotiable fate. The major findings highlighted the promising role of social-ecological system factors in the psychological distress of non-Western adolescents.

## Data availability statement

The raw data supporting the conclusions of this article will be made available by the authors, without undue reservation.

## Ethics statement

The studies involving human participants were reviewed and approved by Institutional Review Board (or Ethics Committee) of the School of Smart Education, Jiangsu Normal University. Written informed consent to participate in this study was provided by the participants’ legal guardian/next of kin.

## Author contributions

YZ and XJ: conceptualization, writing—original draft preparation, and writing—review and editing. XJ: methodology, supervision, and funding acquisition. YZ: validation, formal analysis, investigation, resources, visualization, and project administration. All authors contributed to the article and approved the submitted version.

## Funding

This work was supported by Natural Science Foundation of Chongqing China (cstc2021jcyj-msxmX0973); Jiangsu Provincial Education Science Planning Project of 2022, Special Project (C/2022/01/02).

## Conflict of interest

The authors declare that the research was conducted in the absence of any commercial or financial relationships that could be construed as a potential conflict of interest.

## Publisher’s note

All claims expressed in this article are solely those of the authors and do not necessarily represent those of their affiliated organizations, or those of the publisher, the editors and the reviewers. Any product that may be evaluated in this article, or claim that may be made by its manufacturer, is not guaranteed or endorsed by the publisher.

## Footnotes

^1^ https://www.wjx.cn

## Abbreviations

GFI: Goodness-of-fit index
IFI: Incremental fit index
CFI: Comparative fit index
RMSEA: Root mean square error of approximation
